# Mung Bean Protein Improves Hepatic Metabolic Homeostasis via Gut Microbiota Remodeling

**DOI:** 10.3390/foods14122070

**Published:** 2025-06-12

**Authors:** Kaining Han, Zhuoyao Deng, Guangxin Feng, Tanghao Li, Zhili Wan, Jian Guo, Xiaoquan Yang

**Affiliations:** 1 School of Medicine, Sun Yat-sen University, Guangzhou 510275, China; 2 Laboratory of Food Proteins and Colloids, School of Food Science and Engineering, Guangdong Province Key Laboratory for Green Processing of Natural Products and Product Safety, South China University of Technology, Guangzhou 510641, China

**Keywords:** mung bean protein, digestibility, gut microbiota, metabolites, hepatic metabolic homeostasis

## Abstract

Given the well-documented health benefits of plant proteins, mung bean protein has gained increasing attention as a promising plant-based protein source; however, its biofunctional properties have not been fully recognized. This study aimed to evaluate the hepatic metabolic regulatory effects of dietary mung bean protein in murine models, considering the central role of hepatic metabolic homeostasis in systemic regulation. The results demonstrated that dietary mung bean protein, both native mung bean protein isolate (MPI) and heat-denatured mung bean protein isolate (DMPI), restored hepatic metabolic homeostasis, an effect mediated by bioactive microbial metabolites. Notably, our results demonstrated that heat-induced denaturation of mung bean protein markedly alters its gut microbiota-regulating activity. This was evidenced by the observation that MPI tended to increase the abundance of *Bifidobacterium*, whereas DMPI appeared to promote the growth of Lachnospiraceae_NK4A136_group in mice fed a normal diet. Moreover, both MPI and DMPI increased the abundance of potentially beneficial bacteria, such as *Faecalibaculum*, accompanied by reduced serum total cholesterol (TC) levels and intestinal inflammation in a high-fat diet mouse model. The increased abundance of beneficial bacteria was associated with elevated intestinal short-chain fatty acid (SCFA) levels and restored metabolic levels of nonadecanoic acid, indole derivatives, and bile acid (BA) metabolites. Collectively, our results highlight that mung bean protein promotes hepatic metabolic benefits by orchestrating gut microbiota remodeling and modulating their metabolic outputs.

## 1. Introduction

Accumulating evidence from epidemiologic studies has demonstrated that replacing animal protein with plant protein is associated with lower all-cause and cardiovascular mortality [1,2]. Meanwhile, a series of clinical studies indicated that dietary plant proteins exhibit multiple health benefits, including lowering systolic and diastolic blood pressure, improving blood lipids, and reducing risk factors for cardiovascular diseases [3,4,5]. Mung bean protein, as an emerging source of plant protein, has attracted great attention due to its high nutrient bioavailability [6]. Nevertheless, the health benefits of mung bean protein have long been overlooked and underappreciated. It is well known that the major proteins of mung bean are storage globulins, primarily consisting of the basic 7S type (~3%), vicilin type (8S, ~90%), and legumin type (11S, ~8%). Interestingly, mung bean 8S globulins exhibit high structural similarity and sequence homology with other legume 7S globulins, particularly demonstrating approximately 68% amino acid sequence similarity to the soybean 7S protein [7]. Notably, numerous studies have shown that soybean 7S protein improves blood lipid profiles in animals as well as in humans [8,9]. Our earlier findings further demonstrated that soybean 7S protein exhibited superior efficacy compared to soybean protein in modulating host lipid metabolism [10]. Additionally, our recent study demonstrated that the serum triglyceride-lowering effect of soybean 7S protein is mainly derived from its regulatory effects on gut microbiota and bile acid (BA) homeostasis [11]. Currently, some previous studies have reported that dietary mung bean protein mitigates high-fat diet-induced weight gain, as well as hepatic steatosis, fibrosis, and inflammation [12,13]. However, the limited research is far from sufficient to explain the health-regulating effects of dietary mung bean protein.

It has been established that the gut microbiota influences host metabolism by enhancing energy extraction from food and modifying dietary or host-derived compounds that regulate host metabolic pathways [14]. Particularly, numerous studies have demonstrated that the gut microbiota contributes to the digestion, absorption, metabolism, and modification of dietary proteins, while protein components also influence the structure, function, and metabolism of the microbiota [15]. Specifically, the gut microbiota ferments amino acids derived from dietary proteins, generating a variety of metabolites that are either suspected or known to affect host intestinal physiology, liver function, and peripheral tissues [16]. For example, glycine, threonine, glutamate, and ornithine can be metabolized into acetate, whereas threonine, lysine, and glutamate are involved in butyrate synthesis, with propionate primarily synthesized from threonine by the gut microbiota [17]. It should be emphasized that a high-plant-protein diet ameliorated hepatic lipid accumulation via increased production of microbiota-related 12,13-dihydroxyoctadecanoic acid [18]. The latest research also indicated that soybean 7S protein protected against pressure overload-induced heart failure, potentially through the promotion of short-chain fatty acid (SCFA)-producing gut microbiota and increased intestinal SCFAs [19]. Similarly, mung bean protein has been demonstrated to modulate the secretion of intestinal glucagon-like peptide-1 (GLP-1) and BA metabolism through a gut microbiota-mediated mechanism [12]. Furthermore, a previous study demonstrated that mung bean protein supplementation can promote the proliferation of beneficial bacteria and delay aging by reducing oxidative damage [20]. These studies highlight the critical role of the gut microbiota and their metabolites as indispensable mediators of the health-promoting effects of dietary mung bean protein. Notably, the liver is the primary organ exposed to microbial-derived metabolites, which are crucial for the regulation of its immune and metabolic functions [21]. Nevertheless, the influence of mung bean protein-induced alterations in microbial metabolites on hepatic metabolic regulation has been scarcely investigated.

Recent studies have shown that food processing can lead to protein oxidation and denaturation, which may further impact protein digestibility, as well as the composition and abundance of the gut microbiota and their metabolites [22]. Thermal processing is one of the most widely employed food processing methods, which can have either positive or negative impacts on protein digestibility. The heat treatment of legumes can enhance protein digestibility *in vitro* by deactivating heat-sensitive enzymes, such as protease inhibitors, and altering the protein conformation, thereby increasing the accessibility of proteins to enzymatic breakdown [23]. Our studies also revealed that native soybean 7S protein exhibited digestion resistance to pepsin, whereas boiled protein samples were quickly hydrolyzed into low-molecular-weight oligopeptides (<10 kDa) *in vitro* gastric digestion [24]. Furthermore, our recent study demonstrated that the denatured mung bean protein exhibited improved gastrointestinal digestibility compared to its native counterpart, as determined by *in vitro* simulated gastrointestinal digestion [25]. However, few studies to date have systematically explored how thermally modified mung bean protein digestibility affects the composition and metabolism of the gut microbiota. In the present study, we prepared native state and heat-denatured mung bean protein and systematically investigated the effects of dietary mung bean protein on the gut microbiota composition and metabolism in mice fed either a normal diet or a high-fat diet. Simultaneously, metabolomics analysis was used to explore the possible mechanisms involved in the regulation of hepatic metabolic homeostasis. This study is expected to expand the current understanding of the hepatic metabolic regulatory effects of dietary mung bean protein.

## 2. Materials and Methods

### 2.1. Materials and Chemicals

Mung beans were purchased from a local supermarket in Guangzhou (China). ELISA kits for inflammatory cytokines were purchased from Thermo Fisher Scientific Inc. (Waltham, MA, USA). Acetic acid, propionic acid, butyric acid, and 2-ethylbutyric acid were purchased from Sigma-Aldrich Co. (St. Louis, MO, USA). HPLC-grade methanol was purchased from Merck KGaA (Darmstadt, Germany). All other chemicals used were of analytical grade or better.

### 2.2. Preparation of Mung Bean Protein Isolate

Mung bean protein was extracted from mung beans according to the method described by Brishti et al. with some modifications [26]. Briefly, the mung beans were peeled, freeze-dried, and ground to obtain mung bean powder. The mung bean powder was dispersed in distilled water (1:10, *w*/*v*), and the pH was adjusted to 10.0 with 2 M NaOH. After 2 h of stirring, the solution was centrifuged (8000× *g*, 20 min, 4 °C) and the supernatant was collected. Subsequently, the pH of the supernatant was adjusted to 4.5 with 2 M HCl, and the mixture was then subjected to centrifugation to isolate the precipitate. The obtained precipitate was redissolved in distilled water by adjusting the pH to 7.0 and then dialyzed for 48 h. The dialyzed protein solution was freeze-dried to yield native mung bean protein isolate (MPI). For the preparation of heat-denatured mung bean protein isolate (DMPI), the MPI solution was treated at 121 °C for 15 min, followed by spray drying to obtain DMPI. The protein content of MPI and DMPI was 90.69% and 89.32%, respectively, which was determined by the Dumas method [27]. In the following discussion, the term “mung bean protein” collectively refers to both MPI and DMPI.

### 2.3. Animals and Interventions

Male C57BL/6J SPF mice (4-week-old) were obtained from Beijing Hua Fu Kang Biotechnology Co., Ltd. (Beijing, China). All experimental procedures were approved by the Animal Care and Use Committee of South China University of Technology (No. 20211019-01). The mice were housed in a specific pathogen-free (SPF) environment under controlled conditions (temperature: 22 ± 2 °C, humidity: 50 ± 10%), with a 12 h light/dark cycle, and had ad libitum access to food and water. For the normal diet (10% energy from fat) intervention, the mice were acclimatized for 1 week and randomly assigned to three groups with ten mice each, including the normal control (NC) group, the mung bean protein isolate (MPI) group, and the heat-denatured mung bean protein isolate (DMPI) group. The NC group was administered a standard diet (10% energy from fat) with casein as the sole protein source. The MPI group received a diet in which two-thirds of the casein was substituted with MPI, whereas the DMPI group was provided with a diet in which two-thirds of the casein was replaced by DMPI. The intervention was conducted over a four-week period, during which body weight was recorded weekly. For the high-fat diet (60% energy from fat) intervention, the mice were acclimatized for 1 week and randomly assigned to four groups with ten mice each, including the low-fat diet (LFD) control group, the high-fat diet (HFD) control group, the high-fat diet with MPI (HMPI) group, and the high-fat diet with DMPI (HDMPI) group. The LFD group was provided with a diet containing 10% of total energy from fat, with casein as the sole protein source. The HFD group was fed a diet consisting of 60% of total energy from fat, with casein as the sole protein source. The HMPI group was administered a high-fat diet (60% of total energy from fat) in which two-thirds of the casein was substituted with MPI, whereas the HDMPI group received a high-fat diet (60% of total energy from fat) in which two-thirds of the casein was substituted with DMPI. The intervention was conducted over a five-week period, with body weight recorded twice a week. At the end of the experimental intervention, fresh fecal samples were collected, snap-frozen in liquid nitrogen, and stored at −80 °C for subsequent analysis. The mice were then sacrificed, and serum and tissue samples were collected, snap-frozen in liquid nitrogen, and stored at −80 °C for further analysis.

### 2.4. Measurement of Serum Triglyceride (TG) and Total Cholesterol (TC)

The serum TG and TC were measured using the Rayto Chemray 240 automatic biochemical analyzer (Rayto Life and Analytical Sciences Co., Ltd., Shenzhen, China).

### 2.5. Analysis of 16S rRNA Amplicon Sequencing

Total bacterial DNA was extracted from fecal samples using the method previously reported [28]. The quality of bacterial DNA was assessed using a NanoDrop2000 spectrophotometer (Thermo Fisher Scientific, USA) and agarose gel electrophoresis. The V3–V4 region of the bacterial 16S rRNA gene was amplified using barcoded primer pairs (338F and 806R, Table S1). The PCR amplicons were purified, quantified, and pooled in equal amounts to prepare a sequencing library. Sequencing was performed on an Illumina Nextseq2000 platform (Illumina, San Diego, CA, USA) to generate raw sequencing data. The raw data were further processed through quality control, filtering, assembly, and chimera removal to generate high-quality sequences. The resulting high-quality sequences were clustered into operational taxonomic units (OTUs) using the Uparse algorithm [29]. The taxonomic annotation of representative sequences for each OTU was conducted using the RDP classifier [30] and the SILVA rRNA database [31]. The microbial community diversity was assessed using QIIME. LEfSe analysis was performed to identify differentially abundant taxa among the groups.

### 2.6. Short-Chain Fatty Acid (SCFA) Analysis

The quantification of SCFAs in fecal samples was carried out according to the method we previously described with some modifications [11]. Briefly, fecal samples were weighed and mixed with distilled water at a concentration of 0.2–0.3 g/mL, followed by homogenization with zirconium oxide beads using a KZ-III-F tissue homogenizer (Servicebio, Wuhan, China). The homogenate was then centrifuged (12,000× *g*, 10 min, 4 °C), and the supernatant was collected. An aliquot of 0.3 mL of the sample supernatant was mixed with 150 μL of 0.3 M HCl solution containing 0.3 mg/mL of 2-ethylbutyric acid as an internal standard, along with 50 μL of 0.15 M oxalic acid solution. The mixture was subjected to vortex mixing and then allowed to incubate at room temperature for 30 min. Subsequently, the mixture was centrifuged, and the resulting supernatant was used for gas chromatography (GC) analysis. GC analysis was performed using an Agilent 7890B GC system (Agilent Technologies, Santa Clara, CA, USA) equipped with an HP-INNOWax capillary column (30 m × 0.320 mm × 0.25 μm). Nitrogen served as the carrier gas, and the flow rate was maintained at 1 mL/min. The inlet temperature was maintained at 250 °C, and the oven temperature was programmed as follows: initially set at 100 °C, followed by a linear increase to 180 °C at a rate of 5 °C/min. Each SCFA, including acetate, propionate, and butyrate, was quantified using individual standard curves and reported as µmol/g feces in the final results. A representative gas chromatogram is presented in Figure S1.

### 2.7. Measurement of Inflammatory Cytokines

Colon tissue samples were homogenized in ice-cold PBS and centrifuged at 12,000× *g* for 15 min at 4 °C. The supernatants were collected, and the protein concentration was determined using a bicinchoninic acid (BCA) assay. The levels of TNF-α, IL-1β, IL-6, and IL-10 were quantified using commercially available ELISA kits according to the manufacturer’s instructions. Cytokine concentrations were calculated from standard curves and normalized to the total protein content, expressed as pg/mg protein.

### 2.8. Liver Metabolomics Analysis

Liver tissues were weighed (50 mg) and homogenized with 400 μL extraction solvent (methanol–water = 4:1, *v*/*v*), which contained 0.02 mg/mL L-2-chlorophenylalanine as an internal standard. The homogenized samples were subjected to ultrasonic extraction for 30 min at 4 °C using an ultrasonic cleaner (SBL-10DT, Ningbo Scientz Biotechnology Co., Ltd., Ningbo, China), followed by centrifugation (13,000× *g*, 15 min, 4 °C) to obtain the supernatant. Subsequently, the samples were incubated at −20 °C for 30 min and then centrifuged again. The resulting supernatant was used for LC-MS/MS analysis. LC-MS/MS analysis was performed using a Thermo UHPLC-Q Exactive HF-X system (Thermo Fisher Scientific, Waltham, MA, USA) coupled with a Waters ACQUITY HSS T3 column (1.8 μm, 100 mm × 2.1 mm). The chromatographic conditions were as follows: A phase consisted of 0.1% formic acid in water–acetonitrile (95:5, *v*/*v*), and B phase was composed of 0.1% formic acid in acetonitrile–isopropanol–water (47.5:47.5:5, *v*/*v*/*v*). The flow rate was maintained at 0.40 mL/min, and the column temperature was kept at 40 °C. Mass spectrometry analysis was performed in both positive and negative ion modes, with a mass scan range of 70–1050 *m*/*z*. The ion source temperature was set to 425 °C. The ion spray voltage was set to 3500 V for positive ion mode and −3500 V for negative ion mode.

### 2.9. Statistical Analysis

The data were presented as mean ± standard error of the mean (SEM). For statistical comparisons, differences between two groups were assessed using an unpaired two-tailed Student’s *t*-test, whereas differences among multiple groups were analyzed using one-way analysis of variance (ANOVA) with Tukey’s post hoc test. Statistical comparisons were conducted using GraphPad Prism 8.4.2, with *p* < 0.05 considered statistically significant. Correlation analysis was performed using the Spearman correlation test in R (version 4.4.0), with significance defined as *p* < 0.05.

## 3. Results and Discussion

### 3.1. Regulatory Effects of Mung Bean Protein on Gut Microbiota Composition and Metabolism

**Figure 1:**
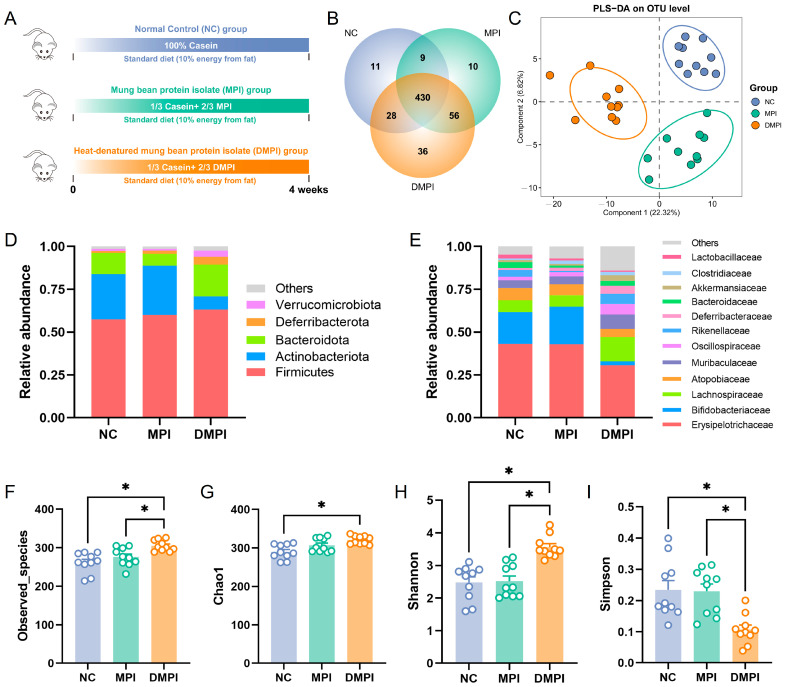
Dietary mung bean protein regulated the composition and diversity of the gut microbiota in the mice fed a normal diet, including the normal control (NC) group, the native mung bean protein isolate (MPI) group, and the heat-denatured mung bean protein isolate (DMPI) group. (**A**) Schematic diagram of the experiment. (**B**) Venn diagram of identified bacterial operational taxonomic units (OTUs). (**C**) Partial least squares discriminant analysis (PLS-DA) of microbial communities based on OTU composition. (**D**,**E**) The relative abundance of the gut microbiota at the phylum level and the family level. (**F**–**I**) The Observed_species, Chao1, Shannon, and Simpson diversity indexes of the gut microbial communities. Data are presented as means ± SEMs (*n* = 10 individuals per group). Significant differences are indicated by * using one-way analysis of variance (ANOVA) with Tukey’s post hoc test.

The effects of dietary mung bean protein on gut microbial composition were first evaluated in mice fed a normal diet (Figure 1A). The body weight dynamics across the experimental groups are shown in Figure S2A, with no significant differences observed. For the composition of the gut microbial community, a total of 580 bacterial OTUs were identified, with 430 OTUs observed across all groups (Figure 1B). The partial least squares discriminant analysis (PLS-DA) at the OTU level revealed that MPI and DMPI significantly altered the gut microbial profiles (Figure 1C). The identified bacteria mainly belong to Firmicutes, Actinobacteriota, Bacteroidetes, Deferribacterota, and Verrucomicrobiota. At the phylum level, the microbial community composition of the MPI group closely resembled that of the NC group. In contrast, the DMPI group exhibited a decreased relative abundance of Actinobacteriota and an increased relative abundance of Bacteroidetes (Figure 1D). At the family level, Erysipelotrichaceae, Bifidobacteriaceae, and Lachnospiraceae were the dominant bacterial families, with other families, including Atopobiaceae, Muribaculaceae, Oscillospiraceae, Rikenellaceae, Deferribacteraceae, and Bacteroidaceae (Figure 1E). Particularly, we observed an increased relative abundance of Lachnospiraceae and a decreased relative abundance of Erysipelotrichaceae and Bifidobacteriaceae in the DMPI group compared with the NC and MPI groups. In addition, the α-diversity analysis of the gut microbial communities showed that dietary DMPI significantly increased the Observed_species, Chao1, and Shannon diversity indexes, while it significantly decreased the Simpson diversity index (Figure 1F–I). These results indicated that thermal denaturation altered the gut microbiota-modulating activity of mung bean protein.

**Figure 2:**
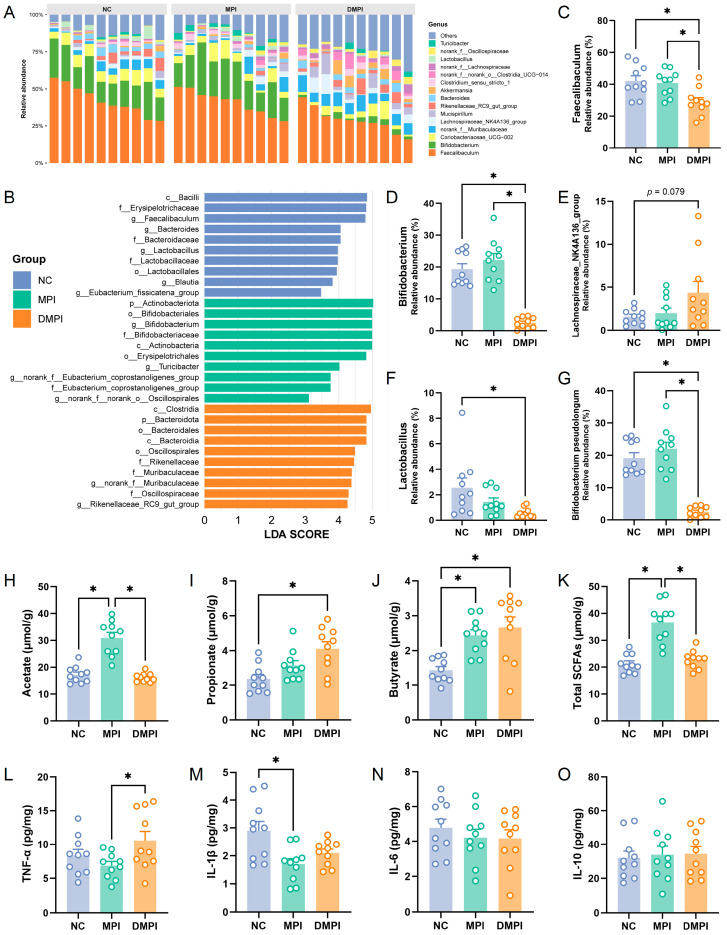
Dietary mung bean protein modulated the gut microbiota composition and metabolic activity in the mice fed a normal diet, including the normal control (NC) group, the native mung bean protein isolate (MPI) group, and the heat-denatured mung bean protein isolate (DMPI) group. (**A**) The overall profile of the gut microbial communities across dietary groups at the genus level. (**B**) LEfSe analysis revealed significantly distinct bacterial taxa among the dietary groups. (**C**–**F**) The relative abundance of *Faecalibaculum*, *Bifidobacterium*, Lachnospiraceae_NK4A136_group, and *Lactobacillus*. (**G**) The relative abundance of *Bifidobacterium pseudolongum* (species level). (**H**–**K**) The concentrations of acetate, propionate, butyrate, and total short-chain fatty acids (SCFAs) in fecal samples. (**L**–**O**) Quantification of inflammatory cytokine levels in colonic tissues. Data are presented as means ± SEMs (*n* = 10 individuals per group). Significant differences are indicated by * using one-way analysis of variance (ANOVA) with Tukey’s post hoc test.

The overall profile of the gut microbial community across the dietary groups at the genus level is shown in Figure 2A. *Faecalibaculum* was the most abundant bacterial genus, followed by *Bifidobacterium*, Coriobacteriaceae_UCG-002, norank_f_Muribaculaceae, Lachnospiraceae_NK4A136_group, and *Mucispirillum*. Concurrently, LEfSe analysis was employed to identify bacterial taxa with statistically significant differences. The results showed that *Faecalibaculum*, *Bacteroides*, *Lactobacillus*, and *Blautia* exhibited significantly higher relative abundances in the NC group, while *Bifidobacterium* and *Turicibacter* were significantly increased in the MPI group (Figure 2B). Particularly, dietary DMPI significantly decreased the relative abundance of *Faecalibaculum* and *Bifidobacterium* compared to the NC and MPI groups (Figure 2C,D). Moreover, dietary MPI increased the relative abundance of *Bifidobacterium* compared to the NC group, with the increase predominantly attributed to *Bifidobacterium pseudolongum* species (Figure 2G), suggesting a potential prebiotic-like effect. In contrast, DMPI elevated the abundance of Lachnospiraceae_NK4A136_group while reducing the *Lactobacillus* level compared to the MPI group (Figure 2E,F), indicating that thermal denaturation altered the interaction between the protein and the gut microbiota. Importantly, *Faecalibaculum rodentium*, an endogenous murine microbiota member that belongs to *Faecalibaculum*, has been shown to generate SCFAs, which contribute to the regulation of protein acetylation and tumor cell proliferation in mice [32]. *Faecalibaculum rodentium* has also been reported to promote epithelial proliferation through a retinoic acid (RA)–eosinophil–IFN-γ-dependent pathway [33]. Additionally, previous studies have demonstrated that *Bifidobacterium pseudolongum* acts as a keystone species, reducing triglyceride levels and suppressing non-alcoholic fatty liver disease [34,35]. These findings suggested that the thermal denaturation of MPI may influence its physiological effects through microbiota-mediated mechanisms.

To investigate the impact of dietary MPI on gut microbial metabolism, we further measured the SCFA concentrations in the fecal samples. The results showed that acetate was the dominant SCFA in the fecal samples, and dietary MPI substantially enhanced the acetate level compared to the NC and DMPI groups (Figure 2H). Meanwhile, dietary MPI significantly increased the concentration of butyrate. In comparison, significantly increased propionate and butyrate levels were observed in the DMPI group (Figure 2I,J). The markedly elevated total SCFAs in the MPI group (Figure 2K) indicated that MPI significantly altered the metabolic activity of the gut microbiota. It is worth noting that *Bifidobacterium pseudolongum* has been reported to be an acetate-producing bacterium that can enhance the host’s antiviral response via the acetate-GPR43 (G-protein-coupled receptor 43) signaling axis [36]. The significantly increased acetate concentration in the MPI group can be attributed to the increased *Bifidobacterium pseudolongum* abundance. Furthermore, it has been reported that Lachnospiraceae_NK4A136_group is a butyrate-producing bacterium that mediates the conversion of acetate to butyrate [37]. The increased butyrate concentration in the DMPI group was closely related to the increased relative abundance of Lachnospiraceae_NK4A136_group. Particularly, previous studies have demonstrated that SCFAs are indispensable in the protection of colonic barrier function and play a crucial role in reducing gut inflammation and maintaining intestinal homeostasis [38]. Subsequently, we further investigated the effects of dietary MPI on the inflammation levels of colon tissue. We observed a significant decrease in the TNF-α levels in the MPI group compared to the DMPI group, as well as a significant reduction in the IL-1β levels in the MPI group relative to the NC group (Figure 2L,M). However, no significant differences were observed in the IL-6 and IL-10 levels among the groups (Figure 2N,O). The reduction in colonic tissue inflammation levels could be associated with the increased production of SCFAs induced by dietary MPI. These findings suggested that MPI and DMPI dietary interventions modulate gut microbiota composition and metabolic activity, potentially contributing to their health-promoting effects. Our earlier study demonstrated that heat treatment enhances the digestibility of mung bean protein [25]. Therefore, our results demonstrated that changes in protein digestibility induced by thermal denaturation may serve as a critical factor influencing the regulatory activity of mung bean protein on the gut microbiota.

### 3.2. Dietary Mung Bean Protein Reduced Serum Lipid Levels and Reshaped Gut Microbiota Composition

**Figure 3:**
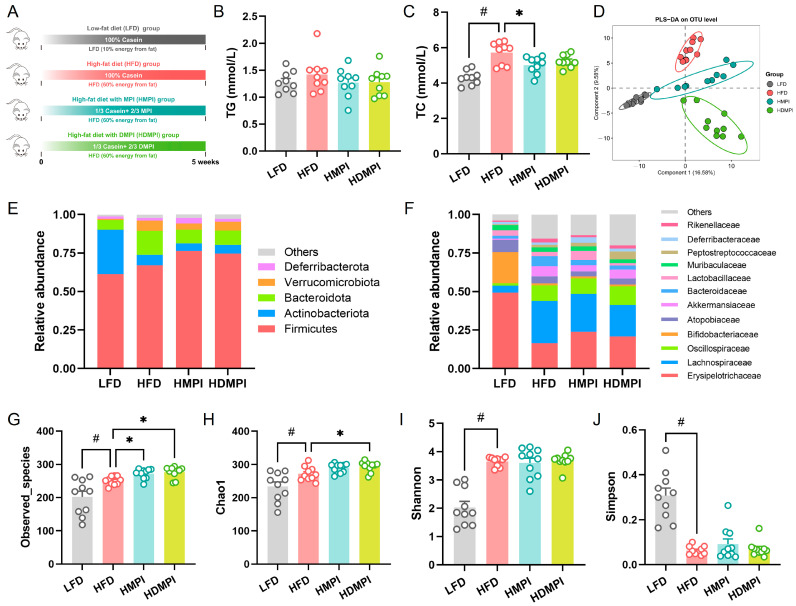
Dietary mung bean protein improved serum lipid metabolism and modulated the gut microbiota composition in the mice fed a high-fat diet, including the low-fat diet (LFD) control group, the high-fat diet (HFD) control group, the high-fat diet with MPI (HMPI) group, and the high-fat diet with DMPI (HDMPI) group. (**A**) Schematic diagram of the experiment. (**B**) Serum triglyceride (TG) level. (**C**) Serum total cholesterol (TC) level. (**D**) Partial least squares discriminant analysis (PLS-DA) of microbial communities based on OTU composition. (**E**,**F**) The relative abundance of the gut microbiota at the phylum level and the family level. (**G**–**J**) The Observed_species, Chao1, Shannon, and Simpson diversity indexes of the gut microbial communities. Data are presented as means ± SEMs (*n* = 9–10 individuals per group). Statistical significance between the LFD and HFD groups was assessed using an unpaired two-tailed Student’s *t*-test (# *p* < 0.05). Comparisons among the HFD, HMPI, and HDMPI groups were conducted using one-way analysis of variance (ANOVA), followed by Tukey’s post hoc test (* *p* < 0.05).

Subsequently, we further evaluated the effects of dietary mung bean protein on the gut microbiota composition and metabolism in a high-fat diet mouse model (Figure 3A). After 5 weeks of dietary intervention, the HFD group exhibited a significant increase in body weight compared to the LFD group, whereas the HMPI and HDMPI groups showed lower body weight than the HFD group (Figure S2B). In addition, the HMPI and HDMPI groups exhibited lower serum TG and TC levels compared to the HFD group (Figure 3B,C). Although a previous study demonstrated that mung bean protein consumption improves glucose and lipid metabolism and may help prevent liver function decline [4], the underlying mechanisms remain to be fully elucidated. Subsequently, we investigated the impact of dietary mung bean protein on the composition of the gut microbiota under high-fat dietary conditions. PLS-DA of OTU-based microbial community profiles showed significant segregation between the dietary intervention groups (Figure 3D), indicating that dietary mung bean protein reshaped the gut microbiota composition. The bacterial composition at the phylum level is shown in Figure 3E. The bacterial communities of the fecal samples were dominated by Firmicutes, followed by Actinobacteriota, Bacteroidetes, Verrucomicrobiota, and Deferribacterota. The high-fat diet significantly reduced the relative abundance of Actinobacteriota and increased the relative abundance of Bacteroidetes compared to the LFD group. In contrast, the HMPI and HDMPI groups showed an increased relative abundance of Firmicutes and a decreased relative abundance of Bacteroidetes compared to the HFD group. At the family level, Erysipelotrichaceae, Lachnospiraceae, Oscillospiraceae, and Bifidobacteriaceae were the dominant families. Specifically, a high-fat diet significantly reduced the relative abundance of Erysipelotrichaceae and Bifidobacteriaceae while increasing the relative abundance of Lachnospiraceae and Oscillospiraceae (Figure 3F). A previous study noted that a high-fat diet leads to an increase in gut microbiota diversity in mice [39]. In the current study, our results showed that a high-fat diet significantly increased the Observed_species, Chao1, and Shannon diversity indexes while reducing the Simpson diversity index compared to the low-fat control diet (Figure 3G–J). Simultaneously, the HMPI and HDMPI groups exhibited a significantly increased Observed_species index, and the HDMPI group showed a significant increase in the Chao1 index compared to the HFD group. These results confirmed that the lipid metabolic regulatory effects of mung bean protein are closely associated with its modulatory impact on the gut microbiota.

**Figure 4:**
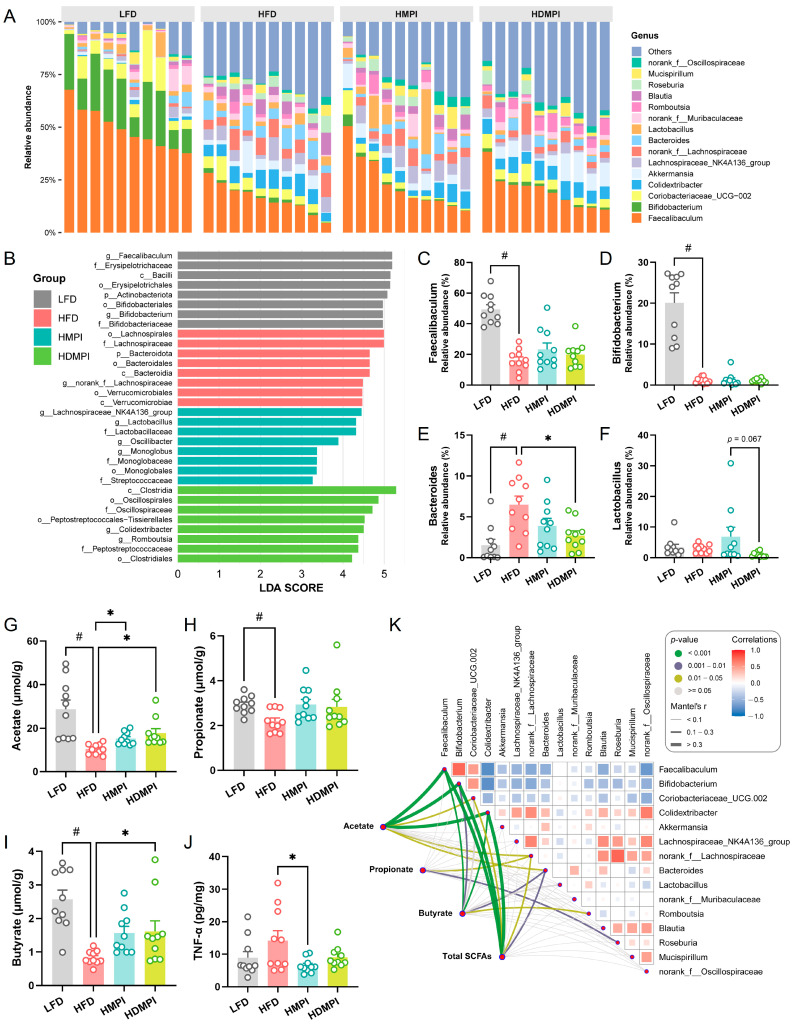
Dietary mung bean protein modulated the gut microbiota composition and metabolic activity in the mice fed a high-fat diet, including the low-fat diet (LFD) control group, the high-fat diet (HFD) control group, the high-fat diet with MPI (HMPI) group, and the high-fat diet with DMPI (HDMPI) group. (**A**) The overall profile of the gut microbial communities across the dietary groups at the genus level. (**B**) LEfSe analysis revealed significantly distinct bacterial taxa among the dietary groups. (**C**–**F**) The relative abundance of *Faecalibaculum*, *Bifidobacterium*, *Bacteroides*, and *Lactobacillus*. (**G**–**I**) The concentrations of acetate, propionate, and butyrate in fecal samples. (**J**) TNF-α level in colonic tissues. (**K**) Mantel test evaluation of the correlations between dominant gut genera and short-chain fatty acid (SCFA) levels. Data are presented as means ± SEMs (*n* = 10 individuals per group). Statistical significance between the LFD and HFD groups was assessed using an unpaired two-tailed Student’s *t*-test (# *p* < 0.05). Comparisons among the HFD, HMPI, and HDMPI groups were conducted using one-way analysis of variance (ANOVA), followed by Tukey’s post hoc test (* *p* < 0.05).

The taxonomic profile of the gut microbiota at the genus level across different dietary intervention groups under a high-fat diet model is shown in Figure 4A. The identified bacterial genera were mainly derived from *Faecalibaculum*, *Bifidobacterium*, Coriobacteriaceae_UCG-002, *Colidextribacter*, *Akkermansia*, Lachnospiraceae_NK4A136_group, norank_f_Lachnospiraceae, *Bacteroides*, *Lactobacillus*, and norank_f_Muribaculaceae. LEfSe analysis revealed that *Faecalibaculum* and *Bifidobacterium* were significantly increased in the LFD group. The Lachnospiraceae_NK4A136_group, *Lactobacillus*, and *Oscillibacter* were significantly enriched in the HMPI group, whereas *Colidextribacter* and *Romboutsia* were significantly elevated in the HDMPI group (Figure 4B). Particularly, we observed that the relative abundance of *Faecalibaculum* was markedly decreased in the HFD group compared to the LFD group. Notably, an increased relative abundance of *Faecalibaculum* was observed in the HMPI and HDMPI groups compared to the HFD group (Figure 4C). In addition, we found that high-fat diets impaired the abundance of *Bifidobacterium*, regardless of the type of dietary protein (Figure 4D). Previous studies have shown that *Faecalibaculum* and *Bifidobacterium* are SCFA-producing bacteria, known to exert beneficial effects on metabolic dysfunction [40,41]. The increased abundance of *Faecalibaculum* in the HMPI and HDMPI groups may contribute to the improvement in serum lipid metabolism. We also noted that dietary mung bean protein counteracted the high-fat diet-induced increase in *Bacteroides* (Figure 4E). Moreover, an increased relative abundance of *Lactobacillus* was observed in the HMPI group compared to the HDMPI group (*p* = 0.067) (Figure 4F). Interestingly, a recent study found that *Oscillibacter*, which was significantly increased in the HMPI group, can metabolize cholesterol in the intestine, thereby helping to reduce cholesterol levels and the risk of cardiovascular diseases [42]. The increased abundance of *Oscillibacter* in the HMPI group may contribute to the significantly reduced serum cholesterol levels compared with the HFD group. A recent study found that Lachnospiraceae_NK4A136_group and *Lactobacillus*, which are associated with SCFA metabolism, might contribute to the Liquiritin apioside-mediated alleviation of colonic inflammation [43]. In addition, *Colidextribacter*, also classified as a beneficial bacterium that promotes SCFA production, has been reported to be associated with fucoidan-mediated alleviation of ulcerative colitis [44]. The increase in these potentially beneficial bacteria in the dietary mung bean protein groups likely contributes to altered SCFA metabolism in the gut. As illustrated in Figure 4G–I, the HMPI and HDMPI groups exhibited elevated acetate, propionate, and butyrate levels compared to the HFD group. Particularly, a significant increase in acetate concentration was observed in the dietary mung bean protein groups compared to the HFD group. The increased SCFA concentration in the dietary mung bean protein groups may be related to reduced colonic inflammation, as evidenced by the significantly reduced level of TNF-α in the HMPI group (Figure 4J).

We further evaluated the correlations between the relative abundance of the main bacterial genera and SCFA concentrations using the Mantel test. The results showed that the acetate and butyrate concentrations were significantly correlated with *Faecalibaculum*, *Bifidobacterium*, and *Colidextribacter* (Figure 4K). Numerous studies have shown that beyond serving as metabolic substrates, SCFAs can function as signaling molecules. SCFA-induced activation of peroxisome proliferator-activated receptor γ (PPARγ) modulates lipid metabolism by enhancing energy expenditure [45]. Therefore, the lipid metabolism-regulating ability of dietary mung bean protein may be related to its modulation of gut microbiota composition and SCFA metabolism. It has been demonstrated that mung bean supplementation can prevent HFD-induced gut microbiota dysbiosis, particularly by enriching certain beneficial bacteria, such as *Bifidobacterium* [46]. In the current study, we further revealed that mung bean protein supplementation can modulate serum lipid metabolism by reshaping the composition and metabolism of the gut microbiota. Unlike the normal diet mouse model, we observed that MPI and DMPI exert similar modulatory effects on the gut microbiota composition and fecal SCFA metabolism in the high-fat diet mouse model, despite exhibiting divergence in some specific indicators. We hypothesized that this might be closely related to the dramatic changes in the gut microbiota caused by the higher fat content in the diet, particularly with a significant decrease in potentially beneficial bacteria, including *Faecalibaculum* and *Bifidobacterium*.

### 3.3. Dietary Mung Bean Protein Improved Hepatic Metabolic Homeostasis

**Figure 5:**
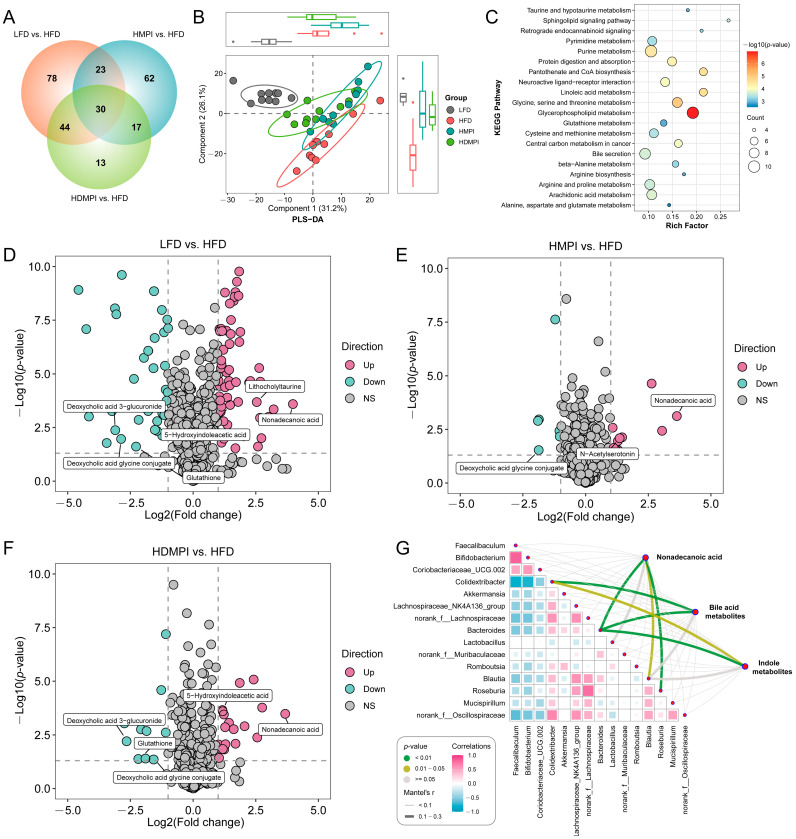
Dietary mung bean protein improved hepatic metabolic homeostasis in the mice fed a high-fat diet, including the low-fat diet (LFD) control group, the high-fat diet (HFD) control group, the high-fat diet with MPI (HMPI) group, and the high-fat diet with DMPI (HDMPI) group. (**A**) Venn diagram of differential metabolites in the liver for LFD vs. HFD, HMPI vs. HFD, and HDMPI vs. HFD. (**B**) Partial least squares discriminant analysis (PLS-DA) based on hepatic metabolites. (**C**) KEGG pathway enrichment analysis of identified metabolites. (**D**–**F**) The differential metabolite profiles for LFD vs. HFD, HMPI vs. HFD, and HDMPI vs. HFD with the screening criteria of *p* < 0.05 and |Log2(Fold Change)| ≥ 1. (**G**) Mantel test evaluation of the correlations between dominant gut genera and hepatic metabolites (nonadecanoic acid, bile acid metabolites, and indole metabolites). *n* = 10 individuals per group.

**Figure 6:**
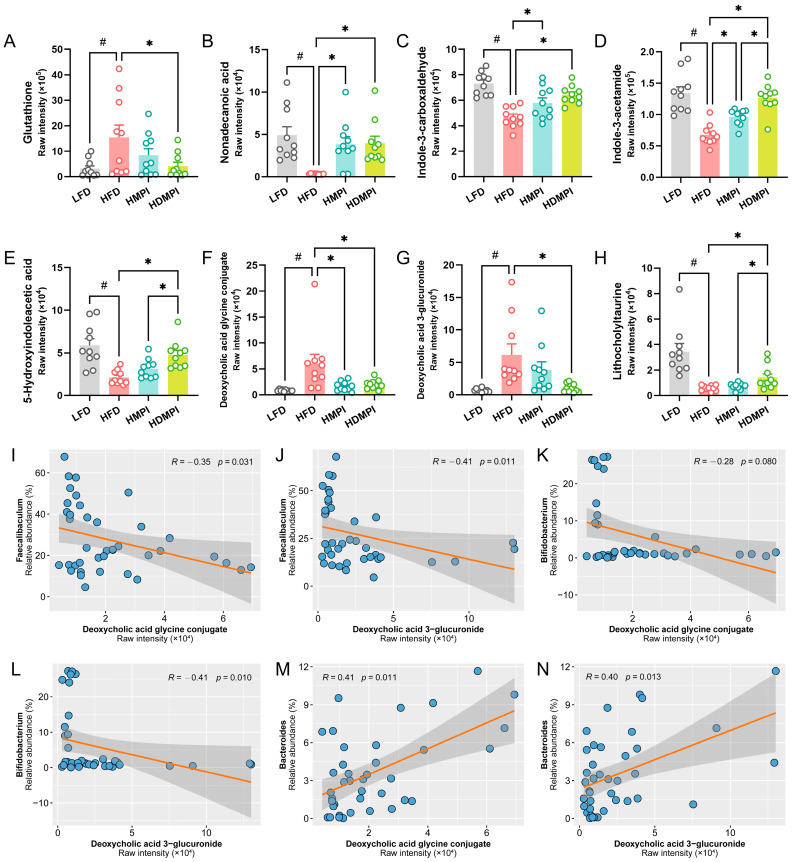
The hepatic differential metabolites across the dietary groups, including the low-fat diet (LFD) control group, the high-fat diet (HFD) control group, the high-fat diet with MPI (HMPI) group, and the high-fat diet with DMPI (HDMPI) group. (**A**) Glutathione level. (**B**) Nonadecanoic acid level. (**C**–**E**) Indole-3-carboxaldehyde, indole-3-acetamide, and 5-hydroxyindoleacetic acid levels. (**F**–**H**) Deoxycholic acid glycine conjugate, deoxycholic acid 3-glucuronide, and lithocholyltaurine levels. (**I**–**N**) Correlation analyses between the relative abundances of *Faecalibaculum* (**I**,**J**), *Bifidobacterium* (**K**,**L**), and *Bacteroides* (**M**,**N**) and the raw intensities of deoxycholic acid glycine conjugate and deoxycholic acid 3-glucuronide, respectively. Data are presented as means ± SEMs (*n* = 10 individuals per group). Statistical significance between the LFD and HFD groups was assessed using an unpaired two-tailed Student’s *t*-test (# *p* < 0.05). Comparisons among the HFD, HMPI, and HDMPI groups were conducted using one-way analysis of variance (ANOVA), followed by Tukey’s post hoc test (* *p* < 0.05). Correlation analysis was performed using the Spearman correlation test, with significance defined as *p* < 0.05 (*n* = 9–10 individuals per group).

To investigate the effects of dietary mung bean protein on hepatic metabolic homeostasis, we systematically analyzed hepatic metabolic profiles using mass spectrometry-based metabolomics. A total of 598 metabolites were identified in the metabolomics analysis. We observed that 175 metabolites were significantly different between the LFD and HFD groups, 132 metabolites were significantly different between the HMPI and HFD groups, and 104 metabolites were significantly different between the HDMPI and HFD groups (Figure 5A). The PLS-DA based on the metabolite profiles showed that the LFD group was clearly separated from all other groups in component 1, and the HFD group was clearly separated from all other groups in component 2 (Figure 5B). These results indicated a significant impact of dietary mung bean protein on the hepatic metabolic profiles. The KEGG pathway enrichment analysis indicated that the identified metabolites were mainly involved in glycerophospholipid metabolism, glycine, serine, and threonine metabolism, purine metabolism, protein digestion and absorption, arachidonic acid metabolism, and bile secretion (Figure 5C). Analysis of differential metabolites between groups revealed that glutathione, deoxycholic acid glycine conjugate, and deoxycholic acid 3-glucuronide levels were significantly increased in the HFD group, while nonadecanoic acid, lithocholyltaurine, and 5-hydroxyindoleacetic acid levels were significantly elevated in the LFD group (Figure 5D). We observed significantly elevated nonadecanoic acid and N-acetylserotonin and significantly reduced deoxycholic acid glycine conjugate in the HMPI group compared to the HFD group (Figure 5E). Moreover, nonadecanoic acid and 5-hydroxyindoleacetic acid were significantly increased, and glutathione, deoxycholic acid glycine conjugate, and deoxycholic acid 3-glucuronide were markedly decreased in the HDMPI group (Figure 5F). Glutathione serves as a key antioxidant and plays a vital role in regulating redox balance and cellular signaling. It protects cells from oxidative damage by neutralizing hydrogen peroxide and eliminating harmful reactive oxygen and nitrogen radicals [47]. A recent study also demonstrated that a high-fat diet promotes glutathione secretion in adipocytes [48]. The significantly increased hepatic glutathione level indicated that a high-fat diet disrupts the oxidative balance of liver cells (Figure 5D and Figure 6A). Interestingly, we observed that nonadecanoic acid was significantly increased in the HMPI and HDMPI groups compared to the HFD group (Figure 5E,F and Figure 6B). Previous studies have revealed that nonadecanoic acid, likely produced by the gut microbiota, was significantly elevated in *Bifidobacterium infantis*-colonized stool samples [49]. Therefore, we hypothesized that gut microbiota regulation mediated by dietary mung bean protein may play a vital role in regulating hepatic metabolism by producing nonadecanoic acid, although the specific role of nonadecanoic acid in maintaining hepatic metabolic homeostasis remains unclear.

Growing evidence indicates that endogenous indole and its derivatives, which are tryptophan metabolites produced by the gut microbiota, exhibit a range of biological activities [50]. It has been shown that indole-3-carboxaldehyde is involved in the anti-obesity effect of *Lactobacillus reuteri* [51]. Moreover, a recent study uncovered that indole-3-carboxaldehyde mitigates the host’s susceptibility to stress by inhibiting neuroinflammation and promoting hippocampal neurogenesis via the aryl hydrocarbon receptor (AhR) signaling pathway [52]. Particularly, previous research has found that indole-3-acetamide can activate hepatic AhR and mediate the protective effects of *Lactiplantibacillus plantarum* against alcoholic liver injury in mice [53]. In addition, 5-hydroxyindoleacetic acid has been shown to effectively alleviate HFD-induced glucose intolerance and obesity while preserving hepatic insulin sensitivity [54]. Interestingly, our results demonstrated that dietary mung bean protein elevated the levels of indole metabolites in the liver compared to the HFD group, including indole-3-carboxaldehyde, indole-3-acetamide, and 5-hydroxyindoleacetic acid (Figure 6C–E). These increased indole metabolites suggested improved hepatic metabolic function. It has been established that the gut microbial dysbiosis caused by HFD disturbs the balance of the gut–liver axis, leading to impaired liver metabolic processes [54]. Thus, we speculated that the gut microbiota changes induced by dietary mung bean protein contribute to the improvement in liver metabolism by mediating the production of indole derivatives.

BAs are initially synthesized from cholesterol in hepatocytes and subsequently modified into secondary BAs by the gut microbiota in the intestinal lumen through a series of modifications, including deconjugation, dehydroxylation, and isomerization [55]. Numerous studies have demonstrated that disrupted BA transport and homeostasis contribute to a variety of liver diseases [56]. Importantly, we noticed that BA-related metabolites, including deoxycholic acid glycine conjugate and deoxycholic acid 3-glucuronide, were significantly elevated in the HFD group, whereas these elevations were attenuated by the mung bean protein intervention (Figure 5E,F and Figure 6F,G). Moreover, the lithocholyltaurine level was significantly increased in the HDMPI group compared to the HFD group (Figure 6H). These results indicated that dietary mung bean protein improved hepatic BA metabolism. It has been reported that dietary mung bean protein modulates host BA metabolism via a gut microbiota-mediated mechanism [12]. Our recent research has also revealed that soybean 7S protein peptides modulate gut microbiota-dependent BA metabolism, primarily reflected in the promotion of fecal secondary BA excretion and hepatic BA synthesis [11]. Notably, these differential BAs are closely associated with secondary BA metabolism, highlighting the critical role of the gut microbiota in maintaining BA homeostasis. Moreover, the Mantel test revealed that the differential BA metabolites were significantly correlated with *Bacteroides* (Figure 5G). Further correlation analysis revealed significant negative correlations between *Faecalibaculum* and both deoxycholic acid glycine conjugate and deoxycholic acid 3-glucuronide, as well as between *Bifidobacterium* and these two metabolites (Figure 6I–L). Additionally, significant positive correlations were observed between *Bacteroides* and both deoxycholic acid glycine conjugate and deoxycholic acid 3-glucuronide (Figure 6M,N). In the current study, dietary mung bean protein decreased the relative abundance of *Bacteroides*, which may contribute to the regulation of BA homeostasis, as certain *Bacteroides* species are known to participate in the deconjugation of primary BAs [57]. It is worth noting that the various biotransformations of BAs carried out by gut bacteria seem to regulate host metabolism through the activation of specific nuclear receptors (NRs) and G-protein-coupled receptors (GPCRs) [58]. Thus, we concluded that mung bean protein-mediated regulation of the gut microbiota may be crucial for improving hepatic BA metabolism homeostasis. Nevertheless, the underlying mechanisms of mung bean protein in modulating hepatic BA synthesis and secretion remain poorly understood and merit further investigation. Furthermore, although both types of mung bean proteins (MPI and DMPI) exhibited similar trends in improving hepatic metabolic homeostasis, certain metabolic indicators showed distinct effects. We speculated that these differences may be closely associated with their distinct modulation of the gut microbiota. Nevertheless, this study has certain limitations, including the lack of in-depth signaling pathway analysis and the relatively short duration of the dietary intervention. Further studies are warranted to elucidate the underlying mechanisms and evaluate the long-term effects of mung bean protein intervention.

## 4. Conclusions

In summary, our study demonstrated that mung bean protein reshaped the composition and metabolism of the gut microbiota in both normal diet and high-fat diet patterns. Notably, we identified that thermal denaturation-induced alteration in protein digestibility serves as a key mechanistic factor regulating the prebiotic activity of mung bean protein on gut microbial communities. Moreover, our results demonstrated that dietary mung bean protein increased fecal SCFA concentrations and effectively improved the hepatic metabolic homeostasis via a gut microbiota-mediated mechanism. Interestingly, a series of beneficial metabolites produced by the gut microbiota, such as nonadecanoic acid, indole-3-carboxaldehyde, and indole-3-acetamide, were significantly elevated in the livers of the mice fed with mung bean protein. Our findings underscore the pivotal role of the gut microbiota in mediating the hepatic metabolic regulatory effects of dietary mung bean protein. Further studies are warranted to elucidate the signaling pathways involved in the response to these gut microbial metabolites. Given its demonstrated hepatic metabolic benefits through microbiota-mediated pathways, mung bean protein holds promise for incorporation into functional food formulations aimed at supporting metabolic health.

## Data Availability

The original contributions presented in this study are included in this article/the Supplementary Materials. Further inquiries can be directed to the corresponding author.

## Supplementary Materials

The following supporting information can be downloaded at https://www.mdpi.com/article/10.3390/foods14122070/s1, Table S1: PCR primer sequences; Figure S1: A representative gas chromatogram of short-chain fatty acids (SCFAs) analysis; Figure S2: Body weight changes of mice during the dietary intervention. (A) Intervention experiment 1, including normal control (NC) group, native mung bean protein isolate (MPI) group, and heat-denatured mung bean protein isolate (DMPI) group. (B) Intervention experiment 2, including low-fat diet (LFD) control group, high-fat diet (HFD) control group, high-fat diet with MPI (HMPI) group, and high-fat diet with DMPI (HDMPI) group.
