# Supplementary material for: Mung Bean Protein Improves Hepatic Metabolic Homeostasis via Gut Microbiota Remodeling
Source: Foods. 2025 Jun 12;14(12):2070. doi: 10.3390/foods14122070 (PMC12192330; doi:10.3390/foods14122070)
Supplement: Supplementary file 1 [file foods-14-02070-s001.zip › foods-3665445-supplementary.pdf]

Mung Bean Protein Improves Hepatic Metabolic Homeostasis via Gut Microbiota Remodeling

Kaining Han [1], Zhuoyao Deng [2], Guangxin Feng [2], Tanghao Li [2], Zhili Wan [2], Jian Guo [2] and Xiaoquan Yang [2,*]

[1]*School of Medicine, Sun Yat-sen University, Guangzhou 510275, China*

[2]*Laboratory of Food Proteins and Colloids, School of Food Science and Engineering, Guangdong Province Key Laboratory for Green Processing of Natural Products and Product Safety, South China University of Technology, Guangzhou 510641, China*

* Correspondence: fexqyang@scut.edu.cn

Table S1. PCR primer sequences

Primer	Sequence
338F	5’-ACTCCTACGGGAGGCAGCAG-3’
806R	5’-GGACTACHVGGGTWTCTAAT-3’

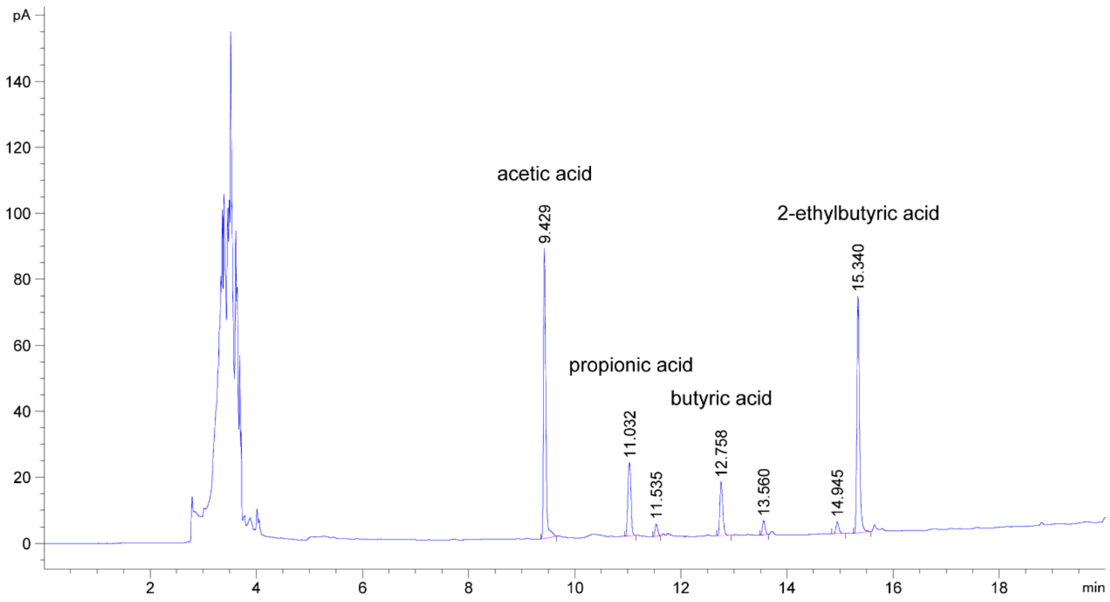

Figure S1. A representative gas chromatogram of short-chain fatty acids (SCFAs) analysis.

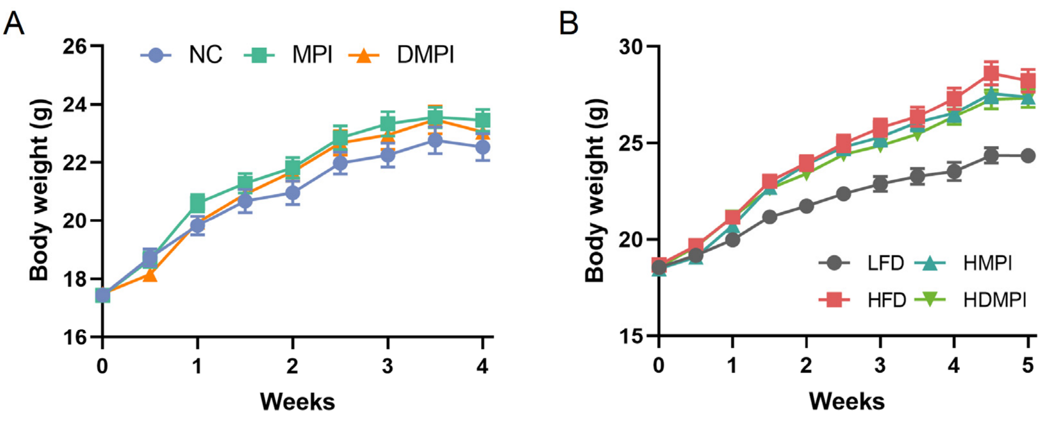

Figure S2. Body weight changes of mice during the dietary intervention. (A) Intervention experiment 1, including normal control (NC) group, native mung bean protein isolate (MPI) group, and heat-denatured mung bean protein isolate (DMPI) group. (B) Intervention experiment 2, including low-fat diet (LFD) control group, high-fat diet (HFD) control group, high-fat diet with MPI (HMPI) group, and high-fat diet with DMPI (HDMPI) group.
